# Early Treatment of Class III Malocclusion: A Boon or a Burden?

**DOI:** 10.5005/jp-journals-10005-1250

**Published:** 2014-08-29

**Authors:** Mohammadi Begum Khan, Arjun Karra

**Affiliations:** Assistant Professor, Department of Orthodontics, Drs Sudha and Nageswara Rao Siddhartha Institute of Dental Sciences, Vijayawada, Andhra Pradesh, India; Assistant Professor, Army College of Dental Sciences, Secunderabad, Telangana India

**Keywords:** Rapid maxillary expansion, Early treatment, Class III malocclusion, Two-phase treatment, Interceptive orthodontics, Skeletal class III malocclusion, Facemask therapy, Mixed dentition stage, Growth modulation, Vertical growth pattern

## Abstract

This article presents a case of class III malocclusion, a female patient aged 8 years treated in early stage of its recognition, i.e. treated in early mixed dentition stage, utilizing orthopedic appliance for its correction, utilizing both rapid maxillary expansion and face mask approach. After the skeletal base correction as part of phase of phase I therapy, a retentive plate was given and patient was asked to report every 6 months for review and monitoring of her growth pattern and phase II treatment planning after the eruption of all permanent teeth.

**How to cite this article:** Khan MB, Karra A. Early Treatment of Class III Malocclusion: A Boon or a Burden? Int J Clin Pediatr Dent 2014;7(2):130-136.

## INTRODUCTION

Angle’s class III malocclusion is one of the malocclusion which shows malrelationship of both the upper and lower jaws in sagittal plane with either maxilla arrested in its sagittal and vertical plane with mandible being prognathic and showing forward rotation or prognathism.^1^ Treatment timing of class III malocclusion has always been controversial in its early stages in young children. Early intervention is needed in children with moderate to severe anterior crossbite and reverse deepbite as sagittal and vertical deficiency of maxilla could contribute to class III malocclusion.^2,3^ Failure of maxilla to grow vertically can result in mandibular overclosure, rotating the mandible upward and forward producing the appearance of mandibular prognathism which could be because of both position and size of the mandible. In such cases, the children can be benefitted by early treatment, because it reduces the psychological burden of facial and dental disfigurement during the formative period of malocclusion.^4^ The etiology of class III malocclusion is multifactorial because of the involvement of genetics, ethnicity, environmental factors and habitual postures. Early treatment of class III malocclusion offers lot of benefit to the patient as the need of the treatment in the permanent dentition will be reduced as the options would be limited to camoufage or surgery.^5^

## CASE REPORT

**Figs 1A to C:**
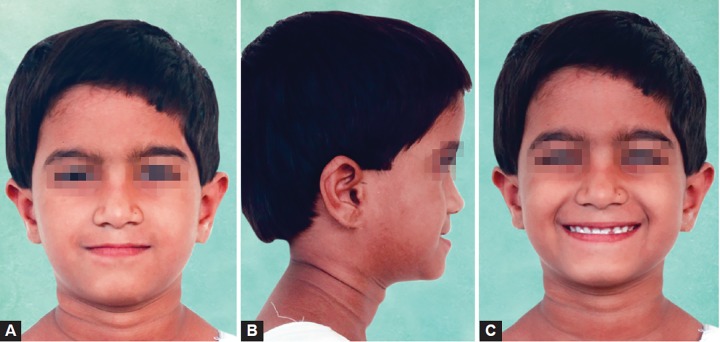
Pretreatment extraoral facial photographs

**Figs 2A to C:**
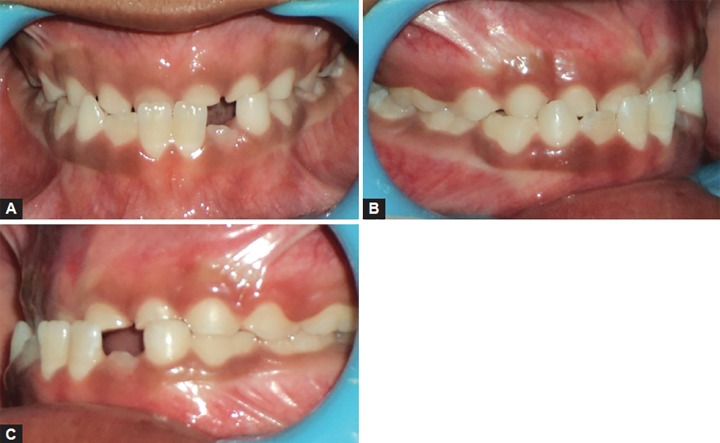
Pretreatment intraoral views showing class III molar relation and anterior crossbite

**Fig. 3:**
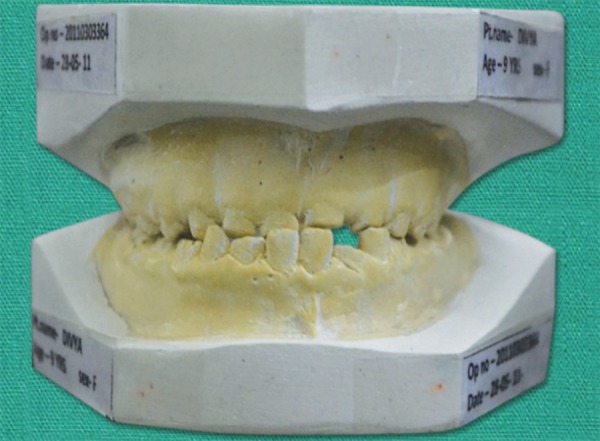
Pretreatment study model

An 8-year-old girl was reported with a chief complaint of poor visibility of the upper front teeth and poor facial appearance. On extraoral examination (Figs 1A to C), her profile was found to be concave and a positive lower lip step was seen, with an appearance of restricted maxillary growth. There was fat midface and prominent appearance of lower lip and chin. The nasolabial angle was right angled and mentolabial sulcus was fat. The smile was unesthetic as the maxillary teeth were having a poor display with more exposure of mandibular teeth. Intraoral examination revealed an early mixed dentition stage with attrited primary central and lateral incisors, completely erupted mandibular central incisors, upper and lower first molars. The anterior crossbite extended from deciduous canine on the right side to the left side. The molar relation on both the sides was class III with reverse overjet of 2 mm and a reverse overbite of 3 mm (Figs 2 and 3).

**Figs 4A and B:**
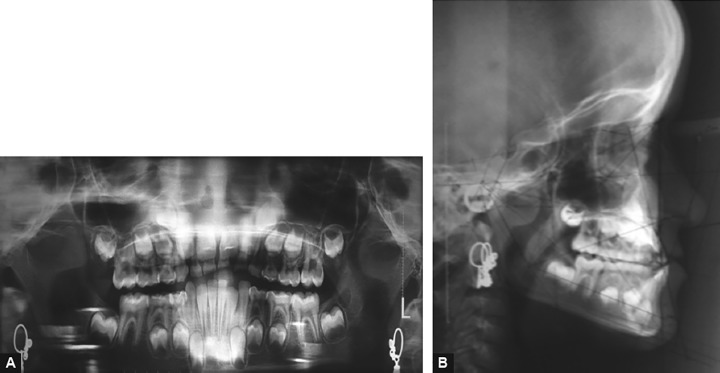
Pretreatment radiographic images: Orthopantomogram (OPG) and cephalogram

**Fig. 5:**
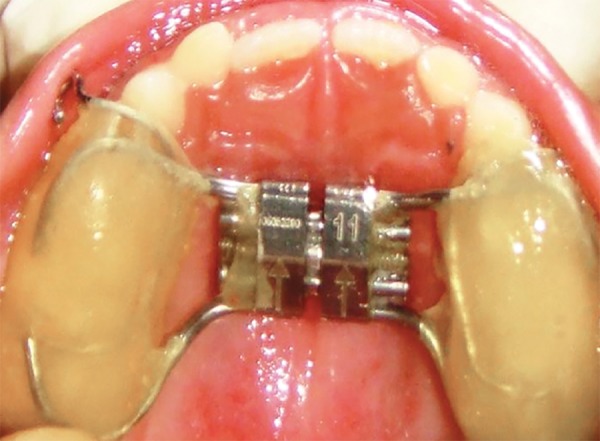
Intraoral view of the appliance showing RME with bondable splint

**Figs 6A and B:**
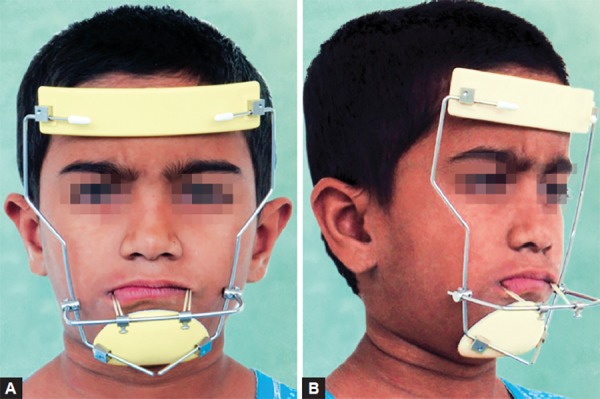
Patient wearing the facemask and elastics

The cephalometric analysis revealed a skeletal class III relationship (ANB 2°, WITTS – 1.5 mm, BO ahead of AO) characterized by maxillary deficiency and mild mandibular prognathism with hypodivergent facial pattern (FMA 24°, SN-GO-GN 28°) the maxillary incisors were proclined moderately (UI-NA 29°), and mandibular incisors were retroclined mildly (LI-NB 30°) to compensate for the skeletal discrepancy (Table 1). The patient was diagnosed as developing skeletal class III due to maxillary deficiency and mandibular protrusion having hypodivergent facial pattern with compensated upper and lower anterior teeth (Figs 4A and B). The appliance selected in this case was Delaire facemask with bondable splint incorporating rapid maxillary expansion (RME) to correct the deficient maxilla. Delaire facemask is a one piece construction with adjustable anterior wire and hooks to accommodate a downward and forward pull of the maxilla with elastics. The intraoral appliance consisted of HYRAX expansion screw incorporated into the acrylic splint on the posterior teeth (Fig. 5). To minimize opening of the bite as the maxilla was repositioned, the protraction elastics were attached near the maxillary canines with a downward and forward pull of 30° to the occlusal plane. The maxillary protraction generally requires 300 to 600 gm of force per side, depending upon the age of the patient. In the present case, elastics that delivered 380 gm (140 z approx) of force per side. Patient was instructed to wear the head gear for 12 to 16 hours a day (Figs 6A and B).

**Table 1:** **Table 1:** Cephalometric data showing pre and post-treatment cephalometric values

*Measurement*		*Norms*		*Pretreatment*		*Post-treatment*	
SNA		82°		77°		81°	
SNB		80°		81°		78°	
ANB		2°		–1.5°		2°	
Wits		0 mm		BO is 3.5 mm ahead AO		AO is 2 mm ahead BO	
SN-GoGn		32°		28°		29°	
FMA		25°		24°		25.5°	
U1 to SN plane		104°		107°		75°	
IMPA		90°		110°		95°	
Nasolabial angle		102°		111 °		98°	
Lower lip to E-line (mm)		–1 mm		2.5 mm		–0.5 mm	

**Figs 7A and B:**
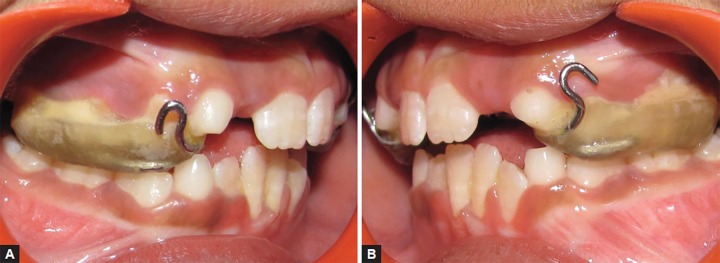
Post-treatment intraoral views with the intraoral bondable expansion splint with hooks showing the desired sagittal and vertical corrections

**Fig. 8:**
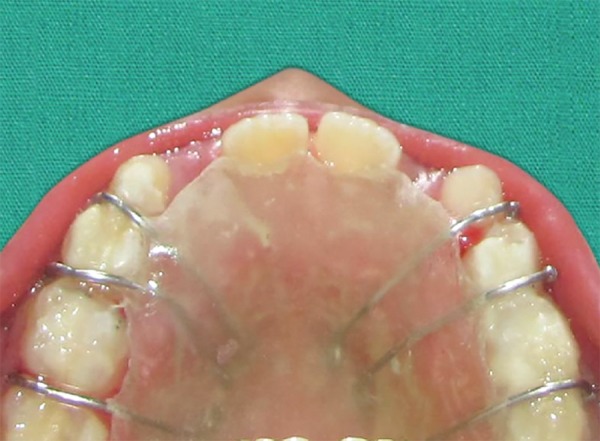
Post-treatment retention plate

The phase I treatment was completed by the over correction of the class III to II molar relation and achieving a positive overjet of about 4.5 mm (Figs 7A and B). The treatment in the phase I was achieved in a time period of about 8 months, post-treatment retentive appliance (Fig. 8) was inserted and the patient was put under observation therapy to monitor the growth pattern till the growth completion gets over.

**Figs 9A to E:**
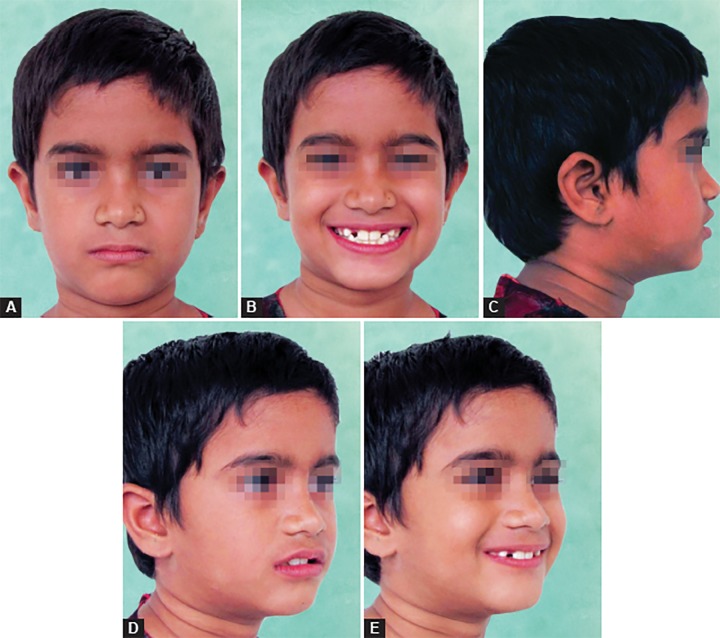
Post-treatment extraoral views: frontal at rest, frontal at smile, profile, sagittal at rest, sagittal at smile

**Fig. 10:**
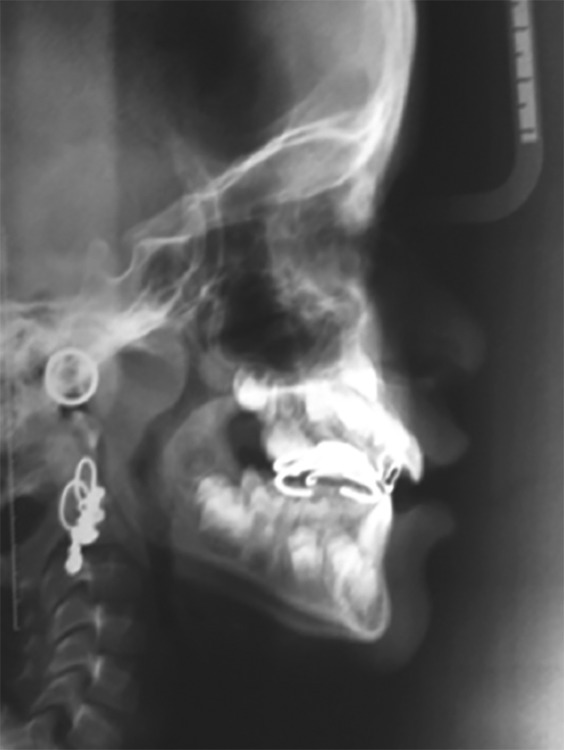
Post-treatment cephalogram showing the skeletal corrections

**Figs 11A to C:**
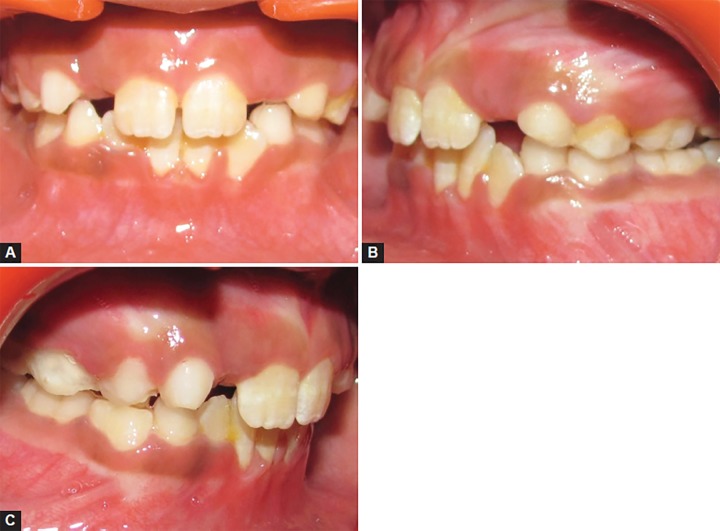
Post-treatment intraoral views after removing the appliance

**Fig. 12:**
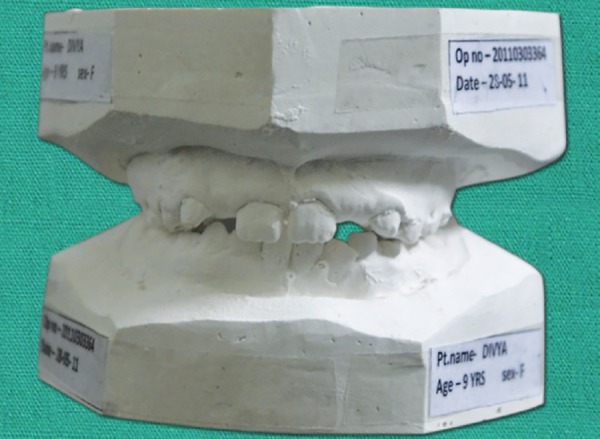
Post-treatment study model

The post-treatment changes in the extraoral appearance (Figs 9A to E) were remarkable with improvement in the overall facial appearance and change in the facial profile from concave to mild convex. The mid face fullness was obvious along with improvement of the lower facial height by about 3 mm indicating downward and backward rotation of the mandible (Fig. 10). The only reason for the over corrections (Figs 11A to C and Fig. 12) was done to compensate the relapse changes which are definitely seen with class III growth modulation cases.

## DISCUSSION

The occurrence of class III malocclusion is believed to be hereditary although environmental factors, such as habits and mouth breathing may play a role. Individuals with class III malocclusion may have combination of skeletal and dentoalveolar components. Protraction facemask therapy has been advocated in the treatment of the class III patients with maxillary deficiency.^6,7^ The positive overjet and overbite at the end of the facemask treatment appears to maintain the anterior occlusion. The dental and skeletal effects of this appliance have been well documented in the literature.^8-12^

The goals of early class III treatment may include the following:

- To prevent progressive irreversible soft tissue or bony changes.
- To improve skeletal discrepancies and provide more favorable environment for future growth.
- To improve occlusal function.
- To provide more pleasing facial esthetics, thus, improving the psychological development of the child.^13^
- Studies have shown that treatment with facemask and or chin cup improves the lip posture and facial appearance.^14^

The question arises as when is the best time to start the protraction facemask treatment. Treatment in the deciduous dentition produces greater skeletal changes than those produced in the mixed dentition stage.^15^ The main objective of early facemask therapy is to enhance forward displacement of the maxilla by sutural growth. It has been shown by Melsen in her histological findings that the midpalatal suture was broad and smooth during the ‘infantile’ stage (8-10 years of age) and the suture became more squamous and overlapping in the ‘juvenile’ stage (10-13 years of age).^16,17^ Moreover, when therapy begins in the early mixed dentition, it seems to induce more favorable changes in the craniofacial skeleton, compared with the same treatment started in the late mixed dentition.^10,18^ Clinically, studies have shown that maxillary protraction was effective in the primary, mixed as well as the early permanent dentitions. The optimal time to intervene a class III malocclusion is at the initial eruption of the maxillary incisors as the circummaxillary sutures are smooth and broad before age 8 years and become more heavily interdigitated around puberty.^16^

Several studies have suggested that a greater degree of anterior maxillary displacement can be found when treatment was initiated in the primary or early mixed dentition. Baccetti et al^10^ examined the differences in early *vs* late treatment in two groups of children treated with bonded maxillary expanders and facemasks. The younger group showed significantly greater advancement of maxillary structures and significantly more upward and forward direction of condylar growth after treatment. A subsequent examination of this sample using Bookstein’s shape-coordinate and tensor analysis confirmed the treatment produced more favorable size and shape changes in the maxilla and the mandible in the early mixed dentition group.^19^

## CONCLUSION

Although the studies have shown that facemask and palatal expansion therapy is an effective method for treatment and earlier intervention might provide a better orthopedic response and is most effective when it begins at an early developmental phase of the dentition (early mixed or late deciduous) rather than during the later stages with respect to untreated class III control groups. Patients treated with RME/ FM therapy in the late mixed dentition, however, still benefit from the treatment but to a lesser degree. Early, treatment produces significant favorable postpubertal modifications in both the maxillary and mandibular structures, whereas late treatment induces only a significant restriction of mandibular growth. The growth treatment response vector (GTRV) ratio calculated during the early permanent dentition will allow the clinician to inform the patients whether malocclusion can be camoufaged by orthodontic treatment or if surgical treatment will be required at a later age.^20^
